# A Mixed-Surface Polyamidoamine Dendrimer for In Vitro and In Vivo Delivery of Large Plasmids

**DOI:** 10.3390/pharmaceutics12070619

**Published:** 2020-07-03

**Authors:** Bhairavi Srinageshwar, Maria Florendo, Brittany Clark, Kayla Johnson, Nikolas Munro, Sarah Peruzzaro, Aaron Antcliff, Melissa Andrews, Alexander Figacz, Douglas Swanson, Gary L. Dunbar, Ajit Sharma, Julien Rossignol

**Affiliations:** 1 College of Medicine, Central Michigan University, Mount Pleasant, MI 48859, USA; srina1b@cmich.edu (B.S.); flore1mv@cmich.edu (M.F.); antcl1ak@cmich.edu (A.A.); andre2mm@cmich.edu (M.A.); figac1am@cmich.edu (A.F.); 2 Program in Neuroscience, Central Michigan University, Mount Pleasant, MI 48859, USA; munro1nm@cmich.edu (N.M.); peruz1st@cmich.edu (S.P.); dunba1g@cmich.edu (G.L.D.); 3 Field Neurosciences Institute Laboratory for Restorative Neurology, Central Michigan University, Mt. Pleasant, MI 48859, USA; 4 Department of Chemistry and Biochemistry, Central Michigan University, Mount Pleasant, MI 48859, USA; clark6bm@cmich.edu (B.C.); johns52k@cmich.edu (K.J.); swans1d@cmich.edu (D.S.); 5 Department of Psychology, Central Michigan University, Mount Pleasant, MI 48859, USA; 6 Field Neurosciences Institute, St. Mary’s of Michigan, Saginaw, MI 48604, USA

**Keywords:** mixed-surface polyamidoamine dendrimers, gene delivery, glial cells, large plasmid, Sox2

## Abstract

Drug delivery to the brain is highly hindered by the presence of the blood–brain barrier (BBB), which prevents the entry of many potential drugs/biomolecules into the brain. One of the current strategies to achieve gene therapy for neurodegenerative diseases involves direct injection of a viral vector into the brain. There are various disadvantages of viral vectors, including limitations of cargo size and safety concerns. Nanomolecules, such as dendrimers, serve as an excellent alternative to viral delivery. In this study, as proof-of-concept, we used a surface-modified dendrimer complex and delivered large plasmids to cells in vitro and in vivo in healthy rats via intracranial injection. The dendrimers were biodegradable by chemicals found within cells and toxicity assays revealed that the modified dendrimers were much less toxic than unmodified amine-surface dendrimers. As mentioned in our previous publication, these dendrimers with appropriately modified surfaces are safe, can deliver large plasmids to the brain, and can overcome the cargo size limitations associated with viral vectors. The biocompatibility of this dendritic nanomolecule and the ability to finely tune its surface chemistry provides a gene delivery system that could facilitate future in vivo cellular reprograming and other gene therapies.

## 1. Introduction

Effective delivery of nucleic acids, such as clustered regularly interspaced short palindromic repeat associated proteins (CRISPR/Cas9), transcription activator-like effectors (TALEs), zinc finger sequences (ZFNs), and small interfering RNA (siRNA), into cells is a critical requirement for cell reprogramming, gene therapy, and vaccine development. In certain cases, only RNA or DNA/plasmids need to be delivered into target cells, while in other cases, concurrent delivery of both DNA and RNA is necessary [1,2,3,4]. Nucleic acids are highly polar compounds with numerous negative charges that restrict their entry into membranes with anionic surfaces. In addition, nucleic acids are readily degraded by nucleases that are ubiquitous in the body. Delivery systems capable of protecting nucleic acids against enzymatic destruction and that facilitate their transport into cells are required for gene-based therapy. For in vitro purposes, the delivery system should be able to bind and package nucleic acid cargo to form a complex, which should subsequently bind to the plasma membrane and become internalized, typically by endocytosis. Once inside the cell, the complex should be released from the endosome and the nucleic acid should be expressed [1]. Delivery to an intact organism also requires that the complex is stable in the bloodstream and extracellular space before being taken up by the target cells.

Several approaches were reported for the delivery of nucleic acid cargo, each with specific advantages and limitations. Notable examples include viral systems, liposomes, and cationic polymers. Viral systems (such as retrovirus, lentivirus, and adenovirus) are the most frequently used in gene therapy clinical trials due to their high transduction efficiency and prolonged gene expression [1]. The major drawbacks of using viral delivery systems include the immunogenicity of viruses, their link to human diseases, limited capacity of cargo size, and complexity of production [5,6,7,8,9,10]. 

Nonviral methods for gene delivery include physical methods, such as the use of voltage or energy to drive DNA into cells or chemicals, such as liposomes and cationic lipids, protein conjugates, linear polymers, dendrimers, or hybrids [11,12]. Compared to viruses, chemical vectors are much simpler systems that are easier to manipulate for the production of safe and reliable pharmaceutical products with batch-to-batch reproducibility. Cationic lipids and liposomes are examples of nonviral delivery systems that are widely used with isolated cells. However, reproducible liposome preparation is challenging and formulations of liposomes with nucleic acids are highly variable and depend on several factors, such as lipid composition, nature of the solvent, and experimental conditions. The physical stability of liposomes may also be compromised by aggregation and coalescence [13,14,15]. 

An exception is the class of nanomolecules known as dendrimers. Synthesis of dendrimers is accomplished through a highly controlled process that leads to the formation of precise, branched nanomolecules that form sharp bands on electrophoresis gels similar to proteins [16]. The molecular weight approximately doubles with generation number (G). A polyamidoamine (PAMAM) dendrimer contains three major regions, namely, a central core, a dendritic or branched interior region, and surface functional groups. In addition to amines, PAMAM dendrimers with surfaces made entirely of other functional groups, such as hydroxyls or carboxyls, are also commercially available. The interior region of a PAMAM dendrimer consists of tertiary amines at the branch points and amide linkages in the branches. Since nucleic acids are anionic, binding and packaging require dendrimers with surface amine groups (pK~9) that are cationic, at physiological pH of ~7. Higher generation amine-surface dendrimers (≥G4) are more effective in packaging larger quantities of nucleic acid and bigger plasmids than lower generations (≤G3).

**Figure 1:**
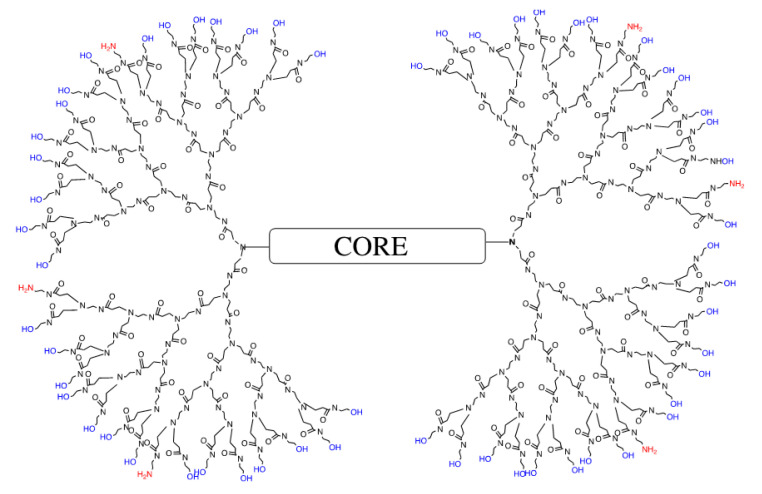
Representation of the G4-90/10 with 90% surface hydroxyl groups and 10% surface amine groups.

One of the most widely studied dendrimers for delivery of nucleic acids in medical applications is the amine-surface PAMAM dendrimer [17,18,19,20,21,22,23,24,25]. While the surface amines play a pivotal role in DNA packaging and cellular uptake, the tertiary amines located within the interior of the dendrimer play an important role as a buffer to help the dendrimer and its bound cargo escape from the endosome after internalization by the cell. For example, a G4 amine-terminated dendrimer is essentially spherical, with 64 amines on its surface and 62 tertiary amines buried within the globular nanomolecule. Although the G4 amine-terminated dendrimer is highly effective in packaging and delivering nucleic acids into cells, it is also more toxic than the lower generation dendrimers due to the high positive charge density from the 64 primary amines on its surface. Therefore, to circumvent this issue, a nanomolecule with a well-defined and reproducible number of surface amines was designed. The current strategy of reducing dendrimer toxicity involves decreasing the number of surface amines by attaching various chemicals, such as polyethylene glycol (PEG), cyclodextrins, glucocorticoids, methyl groups, amino acids, and several others [19,20,21,22,23,24,25]. This strategy requires additional steps of synthesis and purification, complex protection/deprotection steps, is difficult to control, and yields heterogeneous mixtures of dendrimers. The end result is a lack of reproducibility between different batches, a serious obstacle in potential clinical trials [26]. We used a much simpler de novo method, and a well-established PAMAM dendrimer synthesis scheme based on Esfand and Tomalia [27], to reproducibly prepare a G4 mixed-surface dendrimer (G4-90/10), as mentioned in our previous publication [28]. Briefly, we started from the core and synthesized each generation up until a G3 ester, which was then converted into the final product (90% hydroxyl groups, 10% amine groups). This G4-90/10 demonstrated about 58 hydroxyl groups (90%) and 6 amines (10%) on its surface (Figure 1).

Our long-term goal is to treat a variety of diseases through cellular reprograming [29,30,31,32,33,34,35,36]. A specific example is the conversion of glial cells into neuroblasts by introducing the human SOX2 (hSOX2) gene [36]. To that end, we demonstrated, as proof-of-principle, that G4-90/10 successfully introduced nucleic acids into cells in vitro and could deliver a large plasmid, 8.4 kb, containing hSOX2 with a citrine reporter gene, into glial cells in vivo via intracranial injection of the dendrimer–DNA/plasmid complex (dendriplex) in healthy Sprague Dawley rats. 

## 2. Materials and Methods

### 2.1. Synthesis of G4-90/10 and Fluorescent Conjugates

G4-90/10 was synthesized as previously described [28]. The dendrimers were labeled with fluorescein isothiocyanate (FITC; Sigma Aldrich, St. Louis, MO, USA; G4-90/10-FITC) or Cyanine 5.5 (Cy5.5; Lumiprobe, Hunt Valley, MD, USA; G4-90/10-Cy5.5) as previously described [28]. Briefly, the conjugates were purified by dialysis using 3 KDa molecular weight cut-off (MWCO) membranes (Spectrum Labs, Rancho Dominguez, CA, USA) against three changes of 0.9% sodium chloride, followed by deionized water overnight, after which they were lyophilized. The dendrimers were resuspended at the desired concentration in phosphate buffered saline (PBS; pH 7.4) or Hank’s balanced saline solution (HBSS; Gibco, Waltham, MA, USA) for both in vitro and in vivo applications.

### 2.2. Characterization of G4-90/10 and Conjugates

Dendrimers and their conjugates were analyzed by acidic native polyacrylamide gel electrophoresis [16,37] and isoelectric focusing (IEF) [38]. Electrophoresis was performed using a 10% native gel. The gel was run at 100 V for 3.5 h at 4 °C. Dendrimers were also characterized by ^13^C NMR (using Varian Mercury 300 NMR spectrometer: proton decoupled ^13^C NMR spectrum, ^13^C NMR {1H} using a 75 MHz field; modality of acquisition: relaxation delay, 1.000 s; acquisition time, 1.8 s; number of scans, 2000; samples dissolved in D_2_O) and underwent reverse-phase high-performance liquid chromatography (RP-HPLC; Hitachi HPLC system, Tokyo, Japan) using a C18 column (Varian Microsorb-MV; 250 × 4.6 mm, 300 A) attached to a MetaGuard 4.6 mm Microsorb 300 A 5 µ C18 guard column. Mobile phases A and B represented 0.1% aqueous trifluoroacetic acid (TFA) and 0.085% TFA in acetonitrile, respectively. A linear gradient elution of 5–90% B/15 min and a flow rate of 1 mL/min were used. The percentages of surface amines were determined by 2,4,6-trinitrobenzene sulfonic acid assay (TS-28997 Thermo Fisher Scientific, Waltham, MA, USA), using glycine as the standard to prepare the calibration curve.

### 2.3. Dendrimer Degradation

Metabolism of G4-90/10 by various enzymes and physiological chemicals was assessed using acidic native polyacrylamide gel electrophoresis (PAGE) and RP-HPLC.

### 2.4. In Vitro Introduction of G4-90/10 into HEK293 Cells 

Human embryonic kidney cells (HEK293; Cell Biolabs, San Diego, CA, USA) were cultured and a stable cell line was maintained in Dulbecco’s modified eagle’s medium (DMEM; Gibco, Waltham, MA, USA) with 10% fetal bovine serum (FBS; Gibco) and 1% penicillin–streptomycin (P/S; Gibco). We plated 10,000 cells in a 96-well plate containing 0.1 mL media per well. The dendrimers were added to the HEK293 cells at 4 mg/mL (final concentration per well) and incubated for 30 min as previously described [28], after which the existing media were removed and fresh media were added. The nuclei of the cells were stained with Hoechst 33,342 (Sigma Aldrich, Saint Louis, MO, USA). Following incubation, the media were replaced with fresh media and the cells were visualized using an Observer Inverted Microscope (Zeiss, Oberkochen, Germany).

### 2.5. Toxicity of Dendrimer in HEK293 Cells

G4-90/10 was added to the cells as described above and incubated for 15 h. Toxicity was determined using the MTT assay [3(4,5-Dimethylthiazol-2-yl)-2,5-Diphenyltetrazolium bromide]. MTT (Sigma Aldrich, Saint Louis, MO, USA) was added to a final concentration of 12 mM and the cells were incubated at 37 °C for 4 h. Sodium dodecyl sulfate (SDS) was then added and the cells were incubated in the dark at room temperature for 2 h to stop the MTT reaction. When formazan (purple crystals) were observed, absorbance at 570 nm of each well was determined using a SpectraMax M2 (Molecular Devices, San Jose, CA, USA).

### 2.6. In Vitro Introduction of Reporter Plasmid Dendriplex

Reporter plasmids (RPs) were a gift from Dr. Kyle Fink (University of California, Davis, CA, USA). RP1 was 6 kb in size and RP2 was 10 kb in size. Both plasmids contained the mCherry reporter gene expressed under a constitutive promoter. The plasmids were purified from bacteria using Qiagen miniprep kits (Hilden, Germany). The extracted plasmids were quantified using Nanodrop 2000 spectrophotometer (Thermo Fisher Scientific, Waltham, MA, USA) and stored at −20 °C until further use.

Dendriplexes (RP1 dendriplex and RP2 dendriplex) were formed by the reaction of G4-90/10-FITC (10 mg/mL in PBS) with RP1 or RP2 in the dark at room temperature for an hour. Dendriplexe size was determined using dynamic light scattering with a Protein Solutions Dyna Pro (Wyatt Technology Corp, Santa Barbara, CA, USA).

RP1 and RP2 dendriplexes were incubated with HEK293. Final concentrations of dendrimer and DNA per well were 820 µg/mL and 1.3 µg DNA/mL, respectively, at a ratio of dendrimer amines/DNA phosphate (N/P) of 100:1. The cells were periodically analyzed until the mCherry reporter gene expression was observed using an Observer Inverted Microscope (Zeiss, Oberkochen, Germany). Cell viability was also determined using the MTT assay as described above. Cell transfections with Lipofectamine 3000 (Invitrogen, Carlsbad, CA, USA) were used as a positive control.

### 2.7. In Vivo Introduction of hSOX2 Plasmid Dendriplex

Three 20-week-old Sprague Dawley rats (Charles River Laboratories, Wilmington, MA, USA), were used in this study. All procedures associated with animals followed the guidelines of the Institutional Animal Care and Use Committee (IACUC, Memphis, TN, USA) of Central Michigan University (Mt Pleasant, MI, USA; protocol #18–23 approved on 16 August 2018 and #18–07 approved on 24 May 2018). All rats were housed in clear polycarbonate cages at 22 °C with a 12 h light/12 h dark cycle. The animals were given access to food and water ad libitum.

The G4-90/10-Cy5.5 dendrimers were complexed with the hSOX2 plasmid (8.4kb; a gift from Michel Sadelain, Addgene plasmid #23242; Addgene, Cambridge, MA, USA) containing the citrine reporter gene at an N/P ratio of 100:1. Delivery of hSOX2 plasmid (8.4 kb) complexed with G4-90/10-Cy5.5 was by intracranial injection into the striata of healthy rats. A 3 µL injection was delivered at the appropriate coordinates (anterior–posterior or A/P, medial–lateral or M/L, and dorsal–ventral or D/V) using a Hamilton syringe (Hamilton, Reno, NV, USA). The coordinates were A/P: −0.30 mm; M/L: −0.40 mm; D/V: −0.55 mm; and D/V: −0.35 mm. The injection speed was 0.2 µL/min. After each injection, the needle was left in place for 5 min to allow diffusion and then raised slowly. The control rat received the HBSS vehicle. The animals were allowed to recover in their recovery cages. Body temperature was maintained at 37 °C using heating pads. 

The rats were transcardially perfused 72 h after injection with 0.1 M PBS (Sigma Aldrich, Saint Louis, MO, USA), followed by 4% paraformaldehyde (PFA; Sigma Aldrich, Saint Louis, MO, USA) to fix the tissues. The brains were removed and fixed in 4% PFA overnight at 4 °C and then transferred to 30% sucrose solution at 4 °C until they sank (~2–3 days). They were subsequently frozen using 2-methylbutane (Sigma Aldrich, Saint Louis, MO, USA), packed in dry ice, and stored at −80 °C until they were sectioned. The brains were sectioned coronally into 30 µm sections using a cryostat set at −20 °C (Vibratome UltraPro 5000, St. Louis, MO, USA). Glial cells in the brain tissue were visualized by staining with primary antibody against glial fibrillary acidic protein (GFAP) with a prior antigen retrieval step (1 M HCL, pH 0.9). The tissue section was washed 3 × 10 min in 0.01 M PBS and then placed in blocking buffer (10% normal goat serum in 0.1% Triton X-100 in 0.01 M PBS) for 1 h at room temperature. The primary antibody against GFAP (AVES, Tigard, OR, USA) was diluted to 1:5000 in 0.1% Triton X−100 in 0.01 M PBS. Primary antibody was applied to the tissue section and incubated overnight at 4 °C. The tissue section was then washed and reacted with labeled secondary antibody (Alexa Fluor 594 goat anti-chicken Ig at 1:500 dilution) for 1 h at room temperature. Cell nuclei were stained with Hoechst 33,342 (Sigma Aldrich, Saint Louis, MO, USA) at a 1:1000 dilution.

## 3. Results

### 3.1. Synthesis and Characterization of G4-90/10

**Figure 2:**
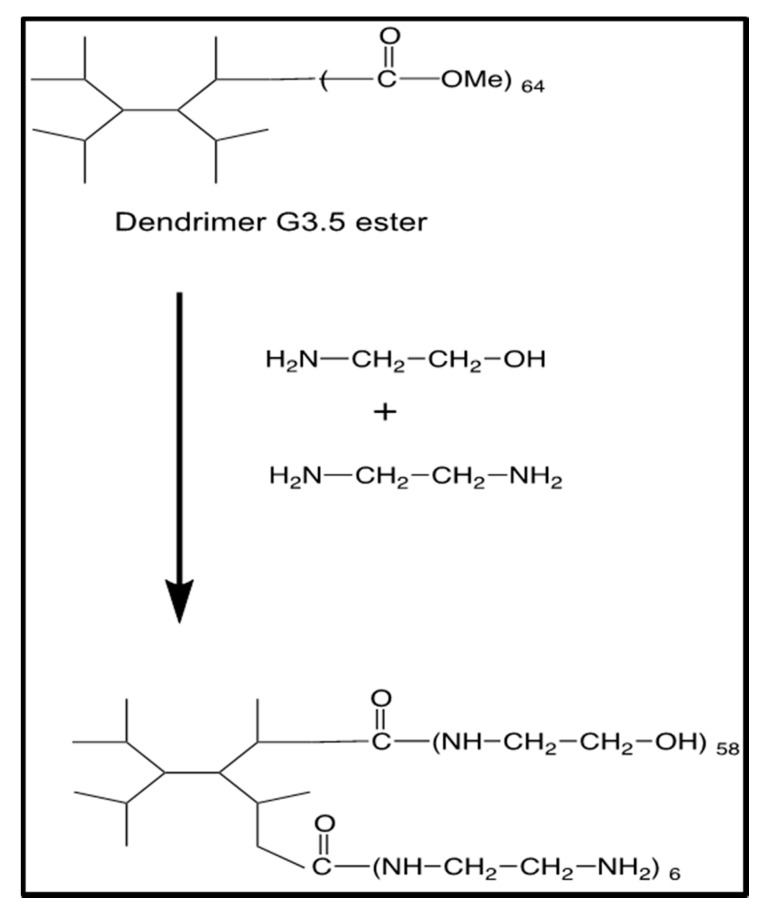
Synthesis of G4-90/10.

We developed a much simpler de novo method for the synthesis of G4-90/10, as previously mentioned in our manuscript [28], based on the original, well-established PAMAM dendrimer synthesis scheme used for synthesis of pure-surface dendrimers [27] (Figure 2). An intermediate G3.5-ester was synthesized as previously reported and subsequently reacted with excess ethanolamine (90 equivalents/ester) and ethylenediamine (10 equivalents/ester) to yield the G4-90/10, with a surface composition of 10% amines and 90% hydroxyl groups.

**Figure 3:**
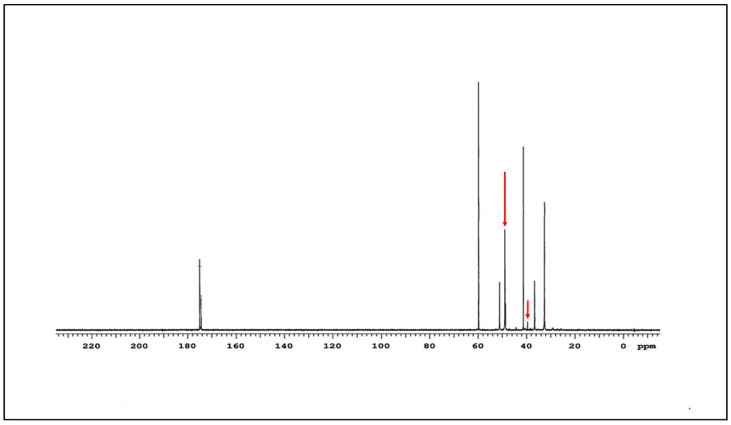
^13^C NMR spectrum of G4-90/10.

The ^13^C NMR spectrum of the G4-90/10 is shown in Figure 3. The amide carbon peaks are at 175 ppm and the rest of the characteristic peaks of G4 PAMAM dendrimer at 32.5, 32.7, 36.6, 41.5, 48.9, and 51.1 ppm are clearly visible. The peaks at around 39.7 and 49.8 ppm (arrow) represent the carbons attached to the surface amine and hydroxyl groups, respectively.

A spectrophotometric amine assay with 2,4,6-trinitrobenzene sulfonic acid was performed on three batches of G4-90/10 with 10% and 30% surface amines. The averages consistently showed 83% of the theoretical values (e.g., 8.3% instead of 10% amines and 25% instead of 30% amines). For the 10% surface amines, this resulted in 5.4 amines instead of 6.4 amines per dendrimer.

PAGE is often used to establish the purity of macromolecules such as proteins, and is applicable for characterization of precise, water-soluble synthetic polymers such as PAMAM dendrimers [16,37]. The purity of the G4-90/10 was similar to the pure-surface dendrimer (G4 amine), as demonstrated by the sharp band observed on the acidic native.

**Figure 4:**
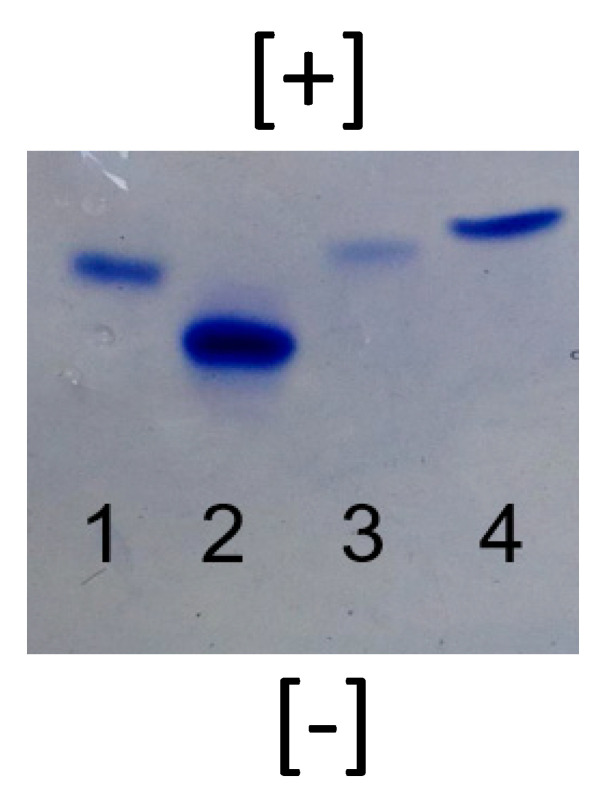
Acidic native PAGE of the nanomolecule. Lane 1 is the mixed-surface G4 dendrimer with 30% surface amines and 70% surface hydroxyl groups, lane 2 is the G4 pure-amine surface dendrimer, lane 3 is the mixed-surface G4 dendrimer with 10% surface amines and 90% surface hydroxyl groups, and lane 4 is the G4 pure hydroxyl surface dendrimer. All dendrimers are at 5 µg/lane.

As expected from the PAGE gel (Figure 4, lane 3), dendrimers migrated based on the density of surface amines. The pure-surface amine dendrimer (100% surface amines) showed the fastest migration (lane 2) followed by the dendrimer with 30% surface amines (lane 1), 10% surface amines (lane 3), and 0% surface amines (pure hydroxyl surface; lane 4). It should be noted that the pure hydroxyl surface dendrimer (lane 4) also migrated in the gel due to its interior tertiary amines, which are protonated under the acidic conditions used for electrophoresis. The isoelectric point (pI) values of different batches of the G4-90/10 were determined using IEF, as previously reported [38], and were found to be around 9.5–10, making the dendrimer a basic “protein mimic” (data not shown).

### 3.2. Degradation of Nanomolecules

**Figure 5:**
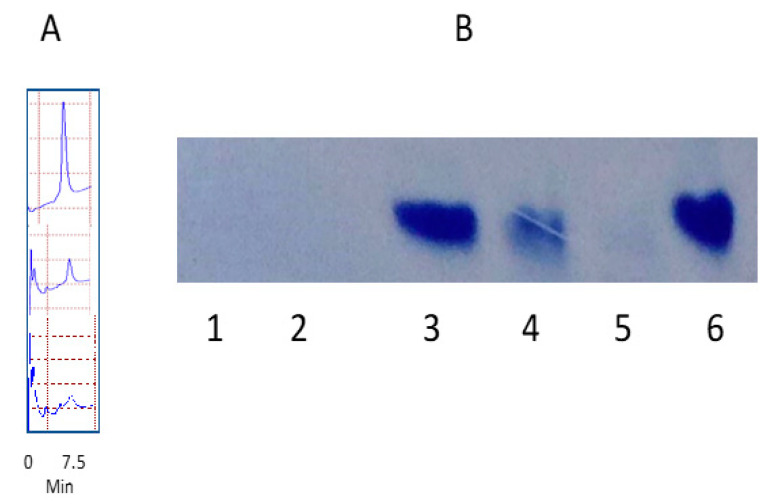
Degradation of G4-90/10 as shown by RP-HPLC (**A**) and acidic native PAGE (**B**). The top HPLC trace is the unreacted G4-90/10, the middle trace is the G4-90/10 reacted with 16 mM H_2_O_2_, and the bottom trace is the dendrimer reacted with 32 mM H_2_O_2_.The dendrimer concentration in all cases was 0.07 mM. In the gel, the dendrimer (0.07 mM) was incubated with 80 mM Fe2+/29 mM H_2_O_2_ (lane 1), 50 mM H_2_O_2_ (lane 2), 5 mM H_2_O_2_ (lane 4), or 500 mM H_2_O_2_ (lane 5) in phosphate buffered saline (PBS) for 24 h at 37 °C. Lanes 3 and 6 are the dendrimer controls (0.07 mM).

We studied the degradation of G4-90/10 using a variety of mammalian enzymes, including lipase, trypsin, papain and lysozyme. There was no significant breakdown of the dendrimer according to the enzymes studied or rat serum, even after 24 h incubation at 37 °C (data not shown). We also looked at the effect of biologically relevant chemicals such as hypochlorous acid (HOCl) and hydrogen peroxide (H_2_O_2_), which are produced by human cells, especially phagocytic cells such as neutrophils and macrophages. RP-HPLC clearly showed the diminished dendrimer peak at 7.5 min and the appearance of new peaks, suggesting breakdown of the dendrimer by 16 mM and 32 mM H_2_O_2_ after 24 h incubation at 37 °C (Figure 5A). The native PAGE showed that H_2_O_2_ as low as 5 mM alone, as well as the Fenton reaction (Fe^2+^/H_2_O_2_), readily degraded G4-90/10 (Figure 5B). These concentrations of H_2_O_2_ occur in the body as the Km for human red blood corpuscles (RBC) catalase, the enzyme that breaks down H_2_O_2_ into water and oxygen, which was reported to be much higher at around 80 mM [39]. HOCl (final concentration 4%, *v*/*v* bleach) did not break down the dendrimer under these conditions (data not shown).

### 3.3. Toxicity of the Nanomolecule

**Figure 6:**
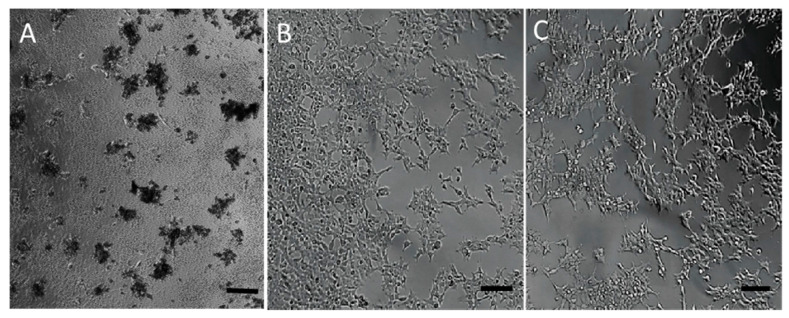
HEK293 cells treated with pure-surface G4 100% surface amine dendrimer (**A**), G4-90/10 (**B**), and untreated control cells (**C**). Scale bar = 100 μm.

As expected, the MTT assays showed that the toxicity of G4-90/10 to HEK293 cells in vitro was dramatically decreased compared to the pure 100% surface amine G4 dendrimer. Cells were treated with relatively high concentrations of the two dendrimers (4 mg/mL final concentration or 280 µM per well). Pure-surface amine-treated cells showed 98.5% cell death compared to the control (untreated) cells. Cells treated with G4-90/10 at the same concentration exhibited only 26.9% death compared to the untreated cells. Morphological differences were also observed between cells treated with the two dendrimers. Cells treated with pure-surface amine dendrimer were shrunken and detached from the culture plate, whereas the cells treated with G4-90/10 were attached and maintained a morphology similar to that of the control cells after 15 h of dendrimer exposure (Figure 6). Similar toxicity profiles were also obtained with neurons (data not shown).

### 3.4. Dendriplex Formation

**Figure 7:**
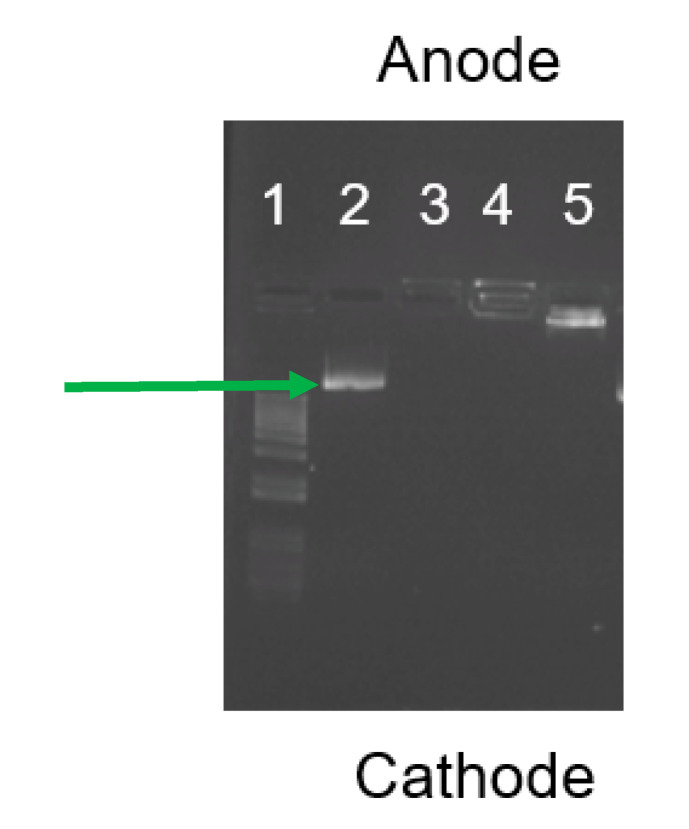
Agarose gel electrophoresis shows the difference in migration of the RP2 when complexed at different N/P ratios, such as 100:1 (lane 3), 10:1 (Lane 4), and 1:1 (lane 5). Lanes 1 and 2 represent the 100 bp ladder and free plasmid (arrow), respectively. The absence of free plasmid bands in lanes 3, 4, and 5 shows that the RP2 is complexed with G4-90/10.

Agarose gel electrophoresis was used to ensure complete complex formation of the DNA/plasmid. These studies showed that N/P ratios of 1:1, 10:1, and 100:1 were able to form complexes with the plasmid. However, the 100:1 ratio was found to form complexes with all of the plasmid, compared to the 1:1 and 10:1 ratios which showed only faint plasmid bands just below the well, indicating negligible amounts of free plasmid available; this was absent in well 3, corresponding to the 100:1 complex (Figure 7). Dynamic light scattering showed that the average hydrodynamic radius of complexes formed between the 10 kb RP and G4-90/10 at N/P of 100:1 was ~135 nm.

### 3.5. In Vitro Uptake of G4-90/10

**Figure 8:**
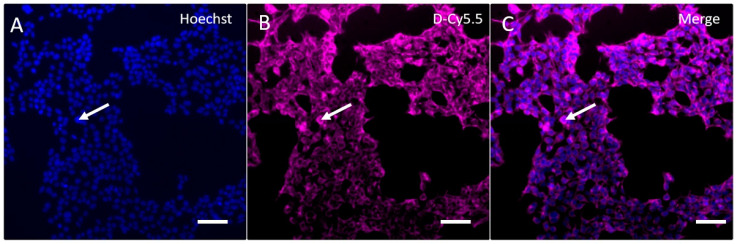
Uptake and retention of G4-90/10-Cy5.5 by HEK293 cells. Cell nuclei labeled with Hoechst 33,342 (arrow) are shown in (**A**), (**B**) shows the uptake of G4-90/10-Cy5.5 dendrimers by HEK293 cells (arrow), and (**C**) is the merged images of the Hoechst and G4-90/10-Cy5.5 staining, showing that G4-90/10-Cy5.5 are taken up by the HEK293 cells. Scale bar = 100 μm.

Fluorescence microscopy showed that the G4-90/10-Cy5.5 was successfully taken up by HEK293 cells following incubation for 30 min at a final concentration of 4 mg/mL, as shown by the localization of Cy5.5 in cells; Hoechst staining shows the cell nuclei (Figure 8).

The image shown in Figure 8 reveals that the transfection efficiency (number of cells stained with Cy5.5/number of nuclei stained with Hoechst) was ~100%.

### 3.6. In Vitro Introduction of RP1 and RP2 and Toxicity

**Figure 9:**
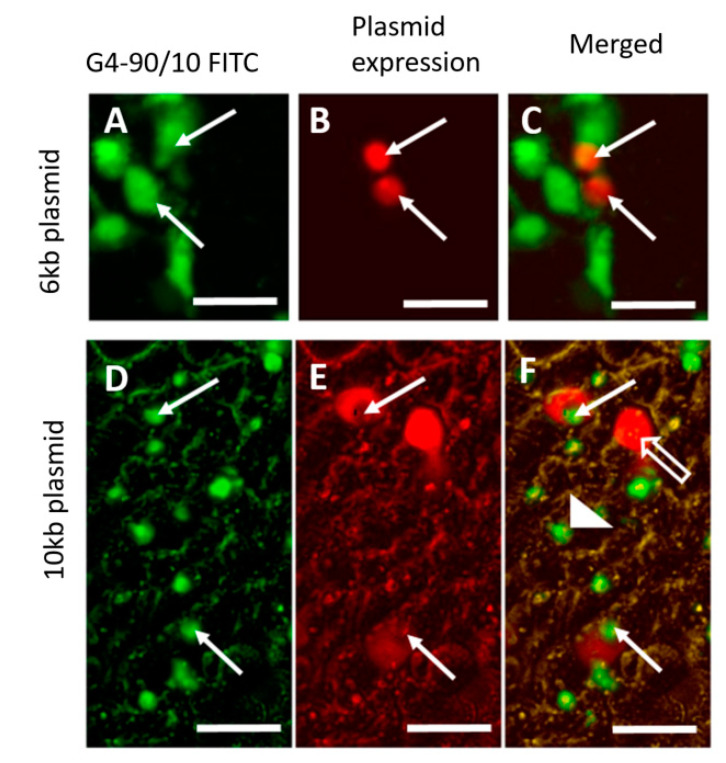
Images (**A**,**D**) show that the dendriplexes formed with G4-90/10-FITC and RP1 are taken up by the HEK293 cells; images (**B**,**E**) show mCherry expression from RP1 and RP2; images (**C**,**F**) (solid arrow) show colocalization between the G4-90/10-FITC and the mCherry. The open arrow and the arrowhead in image F show mCherry expression but exocytosed G4-90/10-FITC and G4-90/10-FITC delivery before mCherry is expressed, respectively (Scale bar: 100 μm).

Fluorescence microscopy showed that the G4-90/10 successfully delivered the 6 kb and 10 kb plasmids to HEK293 cells at an N/P ratio of 100:1 and expressed the mCherry reporter gene (Figure 9). The solid arrows in panels A and D (Figure 9) show the FITC fluorescence exhibited by the dendrimer component of the dendriplex carrying RP1 and RP2, respectively. The solid arrows in panels B and E show the mCherry fluorescence establishing expression of the RP1 and RP2 plasmids, respectively. Colocalization of the FITC fluorescence from the dendrimer and the red fluorescence from the mCherry in the cytoplasm revealed that the dendriplexes were taken up by the cells and the mCherry reporter gene was expressed after 14 h incubation (solid arrows; images C and F). The open arrow in image F shows a cell expressing the mCherry protein without any green fluorescence from the dendrimer, indicating that the dendrimer was exocytosed. The arrowhead shows a cell where the dendriplex was inside the cell, but before mCherry was expressed. The images shown in Figure 9 indicate that the expression efficiency (number of expressing mCherry/number of cells stained with G4-90/10-FITC) was about 33%.

Comparing the toxicity of the complex formed with G4 100% pure-surface amine dendrimer and G4-90/10, only 33% of the cells treated with the pure-amine surface dendriplex survived compared to 97% survival with the G4-90/10 dendriplex. Similar results were obtained with the 6 kb RP1 plasmid (results not shown).

### 3.7. In Vivo Transfection

**Figure 10:**
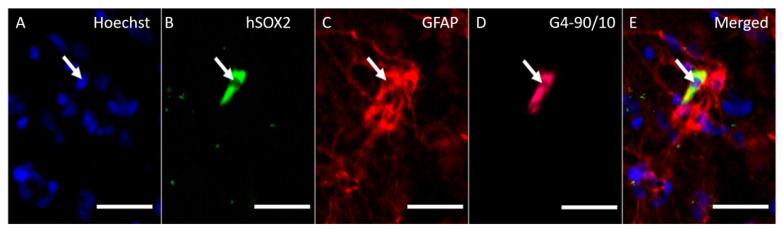
Image (**A**) shows cells labeled with Hoechst; image (**B**) shows citrine expression from the hSOX2 plasmid; image (**C**) shows glial fibrillary acidic protein (GFAP) expression; image (**D**) shows fluorescence from G4-90/10-Cy5.5; image (**E**) is the merge between all images, confirming that the G4-90/10-Cy5.5 dendrimers successfully delivered the large hSOX2 plasmid in vivo and were successfully taken up by the glial cells 72 h post-injection (scale bar: 100 μm).

Intracranial administration of the dendriplexes showed colocalization between hSOX2 (Figure 10B), GFAP (Figure 10C), and Cy5.5 (Figure 10D), demonstrating successful uptake by the glial cells expressing the citrine reporter gene; Hoechst staining was used to identify cells (Figure 10A) (Figure 10).

## 4. Discussion

Our research focus is on the delivery of small molecule drugs and macromolecules into the brain for the treatment of a variety of diseases. One of our specific goals is to reprogram a few glial cells in the brain into functional neurons for treatment of neuronal loss in neuropathological conditions, such as stroke. Reprogramming endogenous cells by inducing expression of one or more transcription factors is a promising alternative to transplantation-based therapy. A variety of somatic cells were successfully reprogrammed, including brain astrocytes into neurons, heart fibroblasts into cardiomyocytes, and exocrine cells of the pancreas into insulin-secreting beta-cells [29,30,31,32,33,34,35]. Su and colleagues reported that delivery of SOX2 DNA to less than 10% of the astrocytes present was sufficient to reprogram them into neuroblasts capable of maturing into synapse-forming neurons in the injured adult spinal cord of mice treated with valproic acid [36]. Since this involved reprogramming only, a small percentage of glial cells was required for therapeutic effect and the development of a highly reproducible, nonviral delivery system with reduced toxicity was more important than its transfection ability.

A spectrophotometric amine assay with 2,4,6-trinitrobenzene sulfonic acid, a sensitive reagent for the determination of amino groups in amino acids and proteins, was used to quantitate the number of surface amine groups per dendrimer. Glycine was used to prepare a calibration curve.

Mixed-surface PAMAM dendrimers, such as G4-90/10, possess both amines that can be protonated to give positive charges and hydroxyls that can become deprotonated to give negative charges. Given this, IEF is a useful technique to determine the isoelectric points of mixed-surface dendrimers, similar to proteins [38]. The pI values of amidoethanol surface and tris(hydroxymethyl)amidomethane (TRIS) surface PAMAM dendrimers, both consisting of 100% hydroxyl groups, were reported to be around 9 and 7, respectively [38]. The amidoethanol dendrimers showed a pI > 7 due to their tertiary amines. Lower pI values of the TRIS surface dendrimer are likely due to the presence of some surface carboxyl groups, which occur as a result of ester hydrolysis during synthesis. The pI values of different batches of the G4-90/10 were found to be around 9–10, making the dendrimer a basic “protein mimic” [28]. Basic proteins, such as cytochrome c (a protein that travels along the inner mitochondrial membrane) and histones (proteins that condense DNA), exhibit pI values between 10–11, while some natural antibodies and therapeutic monoclonal antibodies show pI values as high as 9.5. The basic nature of G4-90/10 facilitates its binding to anionic cell membranes as well as DNA. G4-90/10 conjugates with fluorescent dyes also exhibited sharp protein-like bands on acidic native gels [28].

Long-term accumulation of chemicals that cannot be degraded within cells often leads to adverse health effects [40]. Thus, an important concern for clinical applications is the biodegradation of dendrimer-based nanomedicines under physiological conditions. PAMAM dendrimers smaller than G5 are readily excreted by the kidneys, while generations higher than G5 accumulate in the liver and other organs [41]. Proteolytic enzymes, such as trypsin, chymotrypsin, and papain, did not degrade G4-90/10. Lipases that hydrolyze esters in lipid particles also did not break down the dendrimer. There was also no significant breakdown of the dendrimer by the enzymes present in rat serum. However, H_2_O_2_, which is produced by human cells, especially phagocytic cells such as neutrophils and macrophages, was found to be effective in degrading G4-90/10.

Several in vitro and a few in vivo toxicity studies were reported for PAMAM dendrimers [42,43,44,45,46,47]. Studies on the development of zebrafish embryos showed that surface amine PAMAM dendrimers are more toxic than hydroxyl or carboxyl surface dendrimers [46,47]. Although the in vitro and in vivo toxicity levels of PAMAM dendrimers are not necessarily correlated [42], a common feature reported in most of the previous studies is the significantly higher toxicity associated with amine-surface dendrimers compared to dendrimers with hydroxyl or carboxyl surfaces. The high positive charge density on the surface of amine-surface dendrimers (or other cationic polymers), although beneficial for DNA packaging, is not found in nature and results in complex formation by anionic biomolecules such as serum proteins and membranes, resulting in protein cross-linking and aggregation, blood clotting, hemolysis, membrane damage, oxidative stress, apoptosis, and other deleterious effects [42]. Hence, the need is present for a high-generation dendrimer with minimal surface amines sufficient for complexing with DNA, but with an improved safety profile. 

As expected, the MTT assay showed that the toxicity of G4-90/10 to HEK293 cells in vitro was significantly reduced compared to cells challenged with pure-surface amine G4 dendrimer. Most previous studies used much lower dendrimer concentrations (<100 µM). For example, a dose as low as 6 µM G4 amine-surface PAMAM dendrimer killed over 80% of mouse macrophage J774A.1 cells [45]. In addition to dramatically reduced cellular toxicity, all animals treated with G4-90/10 showed normal feeding, movement, and other behavioral features for at least one week before sacrifice. The G4-90/10 readily formed dendriplexes with plasmids, even as large as 10 kb. It should be noted that the final concentration of the dendrimers in the dendriplex was 0.82 mg/mL, compared to toxicity studies performed with dendrimers alone which resulted in 4 mg/mL (Figure 6).

In vitro and in vivo studies showed that the G4-90/10 dendrimer entered cells in spite of a ten-fold reduction in surface amines. In addition, the reduced surface amines did not prevent the dendrimer from forming complexes (dendriplexes) with large DNA plasmids. It is highly possible that the cationic tertiary amines below the dendrimer surface helped to complex the DNA. Most importantly, the dendriplexes were taken up by cells both in vitro and in vivo and the genetic cargo was expressed, suggesting that these surface-modified dendrimers might offer an excellent alternative to viruses for nucleic acid delivery.

## 5. Conclusions

We designed and synthesized a gene delivery nanomolecule with a favorable safety profile that should be applicable for in vivo cell reprogramming. Unlike modification of dendrimer surfaces to reduce or shield cationic amines, we carried out de novo synthesis of the nanomolecule, which yielded a purer product as demonstrated electrophoresis and chromatography. Despite the limited number of surface amines present, the G4 mixed-surface dendrimer was able to complex large plasmids and deliver them to cells in vitro and in vivo, without interfering with their expression. The decreased cationic surface of the dendrimer and its degradation by physiological amounts of hydrogen peroxide increased the safety profile of the nanomolecule, which is important regarding potential use in human clinical applications.
